# Altuvoct: Innovative Medicinal Products Benefit from Innovative Approaches to Regulatory Assessment

**DOI:** 10.3390/life15060848

**Published:** 2025-05-24

**Authors:** Essam Kerwash, Maria Malamatari, John D. Johnston

**Affiliations:** Medicines and Healthcare Products Regulatory Agency (MHRA), 10 South Colonnade, Canary Wharf, London E14 4PU, UK

**Keywords:** haemophilia A, bleeding episodes, efanesoctocog alfa, Altuvoct, the Toulmin scheme, cause-of-effect analysis

## Abstract

Efanesoctocog alfa (Altuvoct; BIVV001) is a fusion protein comprising domains of (i) factor VIII, (ii) the von Willebrand factor, and (iii) IgG1 coupled to two polypeptide linkers. The half-life of efanesoctocog alfa in plasma is about 40 h. The polypeptide linkers are released by thrombin activation, resulting in an active form of efanesoctocog alfa that results in the formation of a fibrin clot. Data from two single-arm ongoing studies were submitted: the XTEND-1 study enrolled 159 subjects aged 12–72 years, and the XTEND-kids study enrolled 74 subjects aged <12 years; all subjects had severe haemophilia A. Single-arm studies are not amenable to conventional statistical analysis of ‘effect of cause’, and so a supplementary analysis was conducted on the basis of ‘cause of effect’, making use of the scheme described by Toulmin coupled to an analysis of causal inference. Overall, the claim that Altuvoct is indicated to treat people aged ≥2 years with severe (and moderate) haemophilia A was considered to be supported by the results of the submitted studies and associated modelling exercises; the benefit–risk evaluation of Altuvoct was found to be positive in the target population.

## 1. Introduction

Coagulation factor VIII is a protein that consists of six domains, A1-A2-B-A3-C1-C2, and that circulates in the blood as an inactive form bound non-covalently to the von Willebrand factor. The factor VIII protein has a half-life of about 12 h in the bloodstream when stabilised by the von Willebrand factor. In response to insult or injury and exposure to thrombin, factor VIII becomes activated by the cleavage and release of the B domain, separates from the von Willebrand factor, and binds to factor IX to set off a chain of reactions that leads to the formation of a fibrin blood clot. Now no longer protected by the von Willebrand factor, the activated factor VIII protein is quickly cleared from the bloodstream [1].

Haemophilia A is an X chromosome-linked disorder of bleeding that occurs mostly in males and is characterised by a deficiency of functional FVIII in the blood. The condition is caused by a variety of mutations of the FVIII gene. The worldwide prevalence of haemophilia A is estimated to be about 1 in 10,000. The severity of the disease is determined by the activity of endogenous FVIII in the plasma. Thus, severe haemophilia A (<1% endogenous FVIII activity [i.e., <1 IU/dL]) accounts for about 40% of all cases of haemophilia A. Subjects with severe disease usually present in early childhood. Subjects with moderate haemophilia A exhibit between 1 and 5% endogenous FVIII activity [i.e., 1 –5 IU/dL], and subjects with mild haemophilia A exhibit between 5 and 40% endogenous FVIII activity [i.e., 5–40 IU/dL] [2].

Those with severe haemophilia encounter frequent episodes of bleeding into major joints, soft tissues, and muscles, either spontaneously or following minor trauma, leading to significant effects on their physical and psychosocial well-being and quality of life. Intracranial haemorrhage can result in disability and is a leading cause of haemorrhagic death in individuals with haemophilia [2].

Even with treatment with exogenous factor VIII, subjects with severe haemophilia A may experience more than 40 episodes of bleeding per year. However, the current management protocol is to administer products with factor VIII activity intravenously, either on demand or as prophylaxis. With prophylactic treatment, annual median rates as low as one episode of bleeding per year have been reported, and, as a result, prophylactic treatment regimens are being adopted as the standard of care in many countries, with episodic treatment as an alternative option. Factor VIII products have been engineered to prolong their half-lives to about 24 h and, thus, reduce administrations from daily to two or three times per week. The mean terminal half-lives of two extended-half-life (EHL) factor VIII products, Eloctate and Adynovate, were compared in the same subjects and found to be 16.1 h and 16.7 h, respectively, which supports twice-weekly administration [3]. In contrast, efanesoctocog alfa (the active substance of Altuvoct) has been developed to have a half-life in plasma of about 40 h and, thus, permits once-a-week-only administration as prophylaxis for episodes of bleeding [4]. Products with other modes of action, such as emicizumab, concizumab, serpinPC, and gene therapies, are also available [5,6].

Efanesoctocog alfa (the active substance of Altuvoct) is a fusion protein consisting of single-chain B-domain-deleted human factor VIII, the Fc domain of human IgG1, the factor VIII-binding D’D3 domain of human von Willebrand factor and two polypeptide linkers. It is understood that the Fc domain, von Willebrand, and polypeptide linker components contribute to a reduction in the rate of clearance and degradation of the complete fusion protein, giving efanesoctocog alfa a half-life in plasma of about 40 h. The IgG1 Fc region binds to the neonatal Fc receptor (FcRn), which, in turn, delays the lysosomal degradation of immunoglobulins, thereby prolonging the plasma half-life of the fusion protein. Moreover, exposure to thrombin causes the release of the polypeptide linkers, resulting in an active form of efanesoctocog alfa that now acts as a cofactor for activated factor IX, promoting the conversion of factor X to activated factor X, the conversion of prothrombin to thrombin, and the formation of a fibrin clot [4].

Two main studies (both single-arm in design) have been submitted to support the claim that Altuvoct reduces the number of episodes of bleeding in subjects with severe haemophilia A [4,7,8]. The XTEND-1 study enrolled 159 subjects aged 12–72 years, and the XTEND-kids study enrolled 74 subjects aged <12 years; all subjects had severe haemophilia A [9]. In this study, we describe a regulatory review of the use of Altuvoct administered once per week as prophylaxis for episodes of bleeding. The ideal for such exposure to prophylaxis would be to result in zero episodes of bleeding.

## 2. Materials and Methods

### 2.1. Bioanalytical Assay

The primary assay for efanesoctocog alfa was an activated partial thromboplastin time-based one-stage clotting assay with Actin-FSL [9].

### 2.2. Initial Pharmacokinetic Data

Preliminary pharmacokinetic data and population pharmacokinetic analyses were obtained from six early-phase clinical studies that enrolled previously treated patients with severe haemophilia A: 16 subjects in a single-ascending-dose study; 38 subjects in a multiple-ascending-dose study; 13 subjects in a sequential single-dose study (exposing subjects to Advate, Adynovi, and efanesoctocog alfa); 5 subjects in a single-dose study of types 2 and 3 von Willebrand disease; and the XTEND-1 and XTEND-kids studies. The plasma activity of factor VIII after the intravenous administration of efanesoctocog alfa was characterised with a one-compartment pharmacokinetic model with linear elimination. Body weight and Asian race were found to contribute to the variability in pharmacokinetics.

Once-weekly dosing of Altuvoct at 50 IU/kg for 4 weeks compared with 50 IU/kg dosing for 26 weeks resulted in minimal accumulation, consistent with a steady state being achieved after the first dose. Dose proportionality was observed for Cmax and AUC0-tau after the administration of between 25 and 65 IU/kg of efanesoctocog alfa. The incremental recovery of efanesoctocog alfa was comparable to that for other approved factor VIII products, such as Advate and Adynovi. The volume of distribution was 31.3–38.3 mL/kg for dosages in the range of 25–65 IU/kg, and the clearance was 0.50 mL/h/kg. The plasma half-life of efanesoctocog alfa was 47.6 h in adults and adolescents. The half-life of efanesoctocog alfa was shorter in children aged <12 years compared with adults owing to the higher clearance. For the cohorts aged <6 years and 6 to <12 years, the mean half-lives were 39.9 h and 42.4 h, respectively. Inhibitor development in response to factor VIII activity was not detected in the studies [9].

### 2.3. Main Efficacy Studies and Data-Generating Process

Two main ongoing studies were submitted to support claims of reducing the number of episodes of bleeding in subjects with severe haemophilia A. However, these studies were not randomised, not blinded, and did not have internal controls. In order to understand the data-generating process, the studies are presented using the Toulmin style of argumentation.

**Figure 1:**
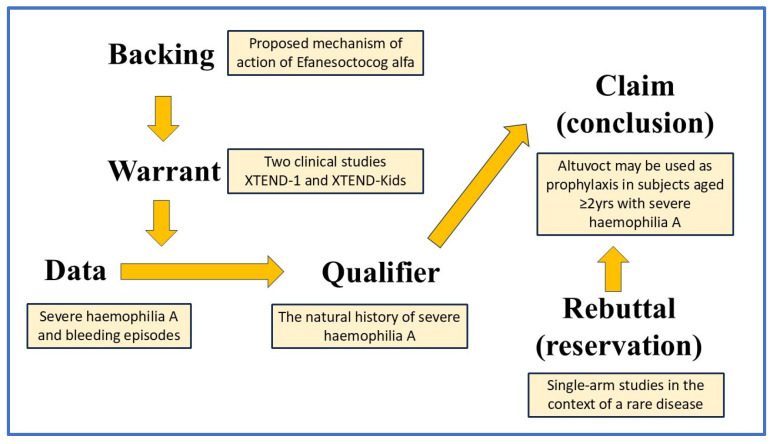
The Toulmin method model, applied to Altuvoct and showing the direction of flow of the argument. A typical argument follows this structure: IF the data is given AND supported by a validated warrant, THEN the claim follows, while also considering any qualifiers and potential rebuttals.

The Toulmin method [10,11], named after the philosopher Stephen Toulmin, is a framework for argumentation that divides an argument into six key elements: data/background information, warrant, claim, qualifier, rebuttal, and backing. As illustrated in Figure 1, the Toulmin method presents arguments in a linear sequence, where each component logically follows from the previous one. This structure aligns well with direction-of-flow analysis, which plays a crucial role in causal inference.

The component parts of the Toulmin method are set out below.

#### 2.3.1. Data/Background Information

Severe haemophilia A is an X-linked disorder caused by genetic mutations of the factor VIII gene that result in <1% activity of plasma factor VIII. People with severe haemophilia A experience recurrent episodes of bleeding.

#### 2.3.2. Warrant

The company submitted two main clinical studies to support claims regarding the use of Altuvoct in subjects with severe haemophilia A. Both studies had a single-arm design. The baseline characteristics and medical histories in both XTEND-1 and XTEND-Kids were typical of a population with severe haemophilia A [8,12].

The XTEND-1 study enrolled subjects who were ≥12 years of age. There were 158 male subjects and 1 female subject, with a mean age of 35 years (range: 12–72 years). In total, 133 subjects in arm A were administered 50 IU/kg of Altuvoct once weekly intravenously as prophylaxis for up to 52 weeks (124 subjects completed the study); 26 subjects in arm B were administered 50 IU/kg of Altuvoct intravenously on demand for 26 months and then transferred to weekly prophylaxis for up to 52 weeks. Altuvoct, administered as once-weekly prophylaxis, maintained the plasma activity of factor VIII at greater than 40 IU/dL for a mean (SD) of 4.1 (0.7) days. The plasma activity of factor VIII was maintained over 10 IU/dL in 83.5% of the adults and adolescent subjects throughout the study. For the XTEND-1 study, the mean annualised bleeding rate in group A was the primary endpoint. Von Drygalski et al. provide more information on the design and conduct of the XTEND-1 study [8].

For the 26 subjects enrolled in the ‘on-demand’ arm for 26 weeks, there were 197 episodes of spontaneous bleeding, 62 episodes of traumatic bleeding, and 9 episodes where the nature of the bleeding was ‘unknown’. For spontaneous annualised episodes of bleeding, only 1 subject did not experience a bleeding episode during this time; 6 subjects had between one and ten episodes; 12 subjects had between ten and twenty episodes; and 7 subjects had more than twenty episodes. For traumatic annualised episodes of bleeding, 24 subjects had up to ten episodes, and 2 subjects had more than twenty episodes. For ‘unknown-type’ annualised episodes of bleeding, 26 subjects had up to ten episodes.

For those on prophylaxis in arm A, subjects experienced up to 5 spontaneous annualised bleeds; up to 10 traumatic annualised bleeds; and up to 10 annualised bleeds of ‘unknown character’ over the median 50 weeks of study. In total, 65% of subjects in the prophylaxis arm of XTEND-1 had zero overall annualised episodes of bleeding. The information is described more fully in Altuvoct UKPAR [9].

The XTEND-Kids study enrolled 74 male subjects between 2 and 12 years of age. The subjects were administered 50 IU/kg of Altuvoct once weekly intravenously as prophylaxis for up to 52 weeks. Altuvoct, administered as once-weekly prophylaxis, maintained the plasma activity of factor VIII at greater than 40 IU/dL for up to 3 days. The plasma factor VIII activity was maintained at over 10 IU/dL for about 7 days. For the XTEND-Kids study, the occurrence of neutralising antibodies against factor VIII (i.e., factor VIII inhibitors) was the primary endpoint. Malec et al. provide more information on the design and conduct of the XTEND-kids study, which is available in [12]. Over the course of the study, there were 42 joint bleeds, 7 muscle bleeds, 5 internal bleeds, and 15 bleeds into the skin or mucosae. In total, 64% of subjects in XTEND-kids had zero overall annualised episodes of bleeding with prophylaxis. The information is described more fully in Altuvoct UKPAR [9].

#### 2.3.3. Backing

The proposed mechanism of action of efanesoctocog alfa (the active substance of Altuvoct) is as a protein with factor VIII function that has been engineered to have a half-life in plasma of about 40 h (compared with the half-life of 12 h of native factor VIII). Efanesoctocog alfa is activated by exposure to thrombin, which sets off a chain of reactions that lead to the formation of a fibrin blood clot.

#### 2.3.4. Qualifier

The natural history of severe haemophilia A is for subjects to experience recurrent episodes of bleeding, either spontaneously or in response to trauma (often mild trauma). In both the XTEND-1 and XTEND-kids studies, treatment-emergent adverse events were mostly ‘mild’ and not considered related to the current product; there were no reports of serious allergies or anaphylaxis or thrombo-embolic events; and treatment-emergent anti-drug antibody responses were not found.

#### 2.3.5. Rebuttal

Two single-arm studies have been conducted in the context of a rare disease. The outcomes of the studies are likely biased because both studies had a single-arm design (i.e., without internal controls), and the XTEND-1 study employed a comparison of the need for Altuvoct as prophylaxis versus on-demand treatment. The long-term maintenance of efficacy beyond the time spans of the two main studies has not yet been established.

#### 2.3.6. Claim

Altuvoct may be used in subjects aged ≥2 years with severe haemophilia A as prophylaxis and to treat episodes of bleeding.

## 3. Results

### 3.1. Cause-of-Effect Analysis

The best way to evaluate the efficacy of a new treatment is by conducting trials that are randomised, blinded, and internally controlled because this form of trial reduces bias, balances covariates, and permits a valid test of significance [13]; this form of trial permits an ‘effect-of-cause’ analysis [14,15]. The XTEND-1 and XTEND-kids studies, however, were not randomised, were not blinded, and did not have internal controls, and so do not lend themselves to an ‘effect-of-cause’ analysis. In order to assess the studies from a clinical perspective, therefore, a supplementary analysis was conducted by causal inference, employing a structural causal model on the basis of ‘cause of effect’, i.e., that an outcome is caused by an identified event [16].

Causality is relative to the model and context. For the XTEND-1 and XTEND-kids studies, the context of the structural causal model is subjects with severe haemophilia A, where ‘normality’ [17] for these subjects would be to experience recurrent episodes of bleeding (spontaneous and/or traumatic) in the absence of intervention. The purpose of intervention is to reduce bleeds, with the ideal being no episodes of bleeding at all.

A structural causal model may be used to establish ‘cause of effect’ by describing the data-generating process coupled to (i) graphical methods, (ii) non-parametric structural equations, and (iii) counterfactual analysis [18]. Thus, we assume that the world is described in terms of variables and their values and that some variables may have a causal influence on others. We create a graphical model of the world to encode causal assumptions by dividing variables into two sets: exogenous variables, whose values are determined by factors outside the model, and endogenous variables, whose values are ultimately determined by the exogenous variables. Acyclic graphs display nodes labelled as either an endogenous or an exogenous variable (exogenous variables form the roots of the model), with a directed edge from variables x to y if y depends on x. The graphical model can now describe what happens in the presence of an external intervention (all medicinal products are exogenous to the recipient) [19,20].

### 3.2. Graphical Model and Associated Non-Parametric Equations

**Figure 2:**
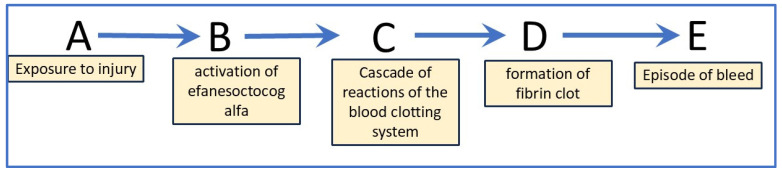
Causal chain representing the formation of a blood clot in study participants who have been administered Altuvoct. Letters A to E: A—exposure to insult or injury, B—activation of efanesoctocog alfa (the active substance of Altuvoct), C—cascade of reactions of the blood-clotting system, D—formation of fibrin clot, and E—episode of bleeding, (i) where B, C and D are mediators for the effect of A on E and (ii) where E is the target quantity that can be measured.

Causal analysis uses directed acyclic graphs (DAGs) to represent causal assumptions. Figure 2 illustrates the DAG for the administration of Altuvoct to individuals with severe haemophilia A. This causal chain consists of exogenous variables (A and B), endogenous variables (C, D, and E), and a set of functions that map the pathway from A to E.

In this model, each node corresponds to an event, while each edge represents a temporal progression from an earlier node to a later one. The DAG captures the relationships between variables in the underlying causal structure: when a variable Y is a product of another variable X in the graph, X is considered a direct cause of Y. Importantly, DAGs are non-parametric; they do not define the functional form or magnitude of the causal relationships but instead provide a qualitative representation [16].

In this scenario, the model comprises four functions, each representing a distinct causal mechanism: *b* = *fb*(*a*,*u*1), *c* = *fc*(*b*,BIVV001,*u*2), *d* = *fd*(*c*,*u*3), and *e* = *fe*(*d*,*u*4). Here, A and efanesoctocog alfa are known exogenous variables, while *u*1–4 denotes exogenous factors excluded from the analysis. The resulting joint probability distribution is expressed as P (*b*,*c*,*d*,*e* | do(*a*)).

### 3.3. Counterfactual Analysis

Causation can be characterised through two key aspects: necessity and sufficiency, that is, some conditions are required for an outcome to occur (necessary), while others are adequate on their own to bring about the outcome (sufficient) [16,18]. Within this framework, three counterfactual probabilities are commonly used to assess causal attribution: the probability of necessity (PN), the probability of sufficiency (PS), and the probability of necessity and sufficiency (PNS). These metrics describe the extent to which an outcome can be attributed to an exposure, under the assumptions of monotonicity and exogeneity.

In the context of subjects with severe haemophilia A, the relevant outcome is the effective clotting of blood (i.e., the absence of bleeding). The assumption of monotonicity posits that changing the exposure variable X from false to true cannot result in a reversal of the outcome Y from true to false. Medicinal products like Altuvoct are considered exogenous to the recipient. The analysis also rests on consistency, meaning treatment effects depend solely on biological responses and not on contextual factors, such as the clinical setting [19,20].

The natural history of severe haemophilia A involves frequent bleeding episodes (both spontaneous and trauma-induced), and patients typically require prophylactic treatment with externally administered factor VIII. There is no known spontaneous remission of the condition without treatment.

The probability of necessity (PN), the probability of sufficiency (PS), and the probability of necessity and sufficiency (PNS) are recognised as follows: let X and Y be two binary variables in a causal model, where X is the input and Y is the output; let x and y stand for the propositions X = true and Y = true, respectively; and let x′ and y′ be their respective complements. In this case, “X= true” represents prophylactic administration of Altuvoct, and “X = false” represents Altuvoct (or any other factor VIII product) not administered; “Y = true” represents zero overall episodes of bleeding, and “Y = false” indicates episodes of bleeding have occurred.

The probability of necessity (PN) is defined as PN = P (Yx = false | X = true, Y = true). This represents the probability that outcome Y (no bleeding) would not have occurred without treatment X, given that both the treatment and outcome did occur. In other words, it quantifies how necessary the treatment was for preventing bleeding. Given the natural course of haemophilia A, in the absence of Altuvoct, patients would almost certainly have continued to bleed. Supporting this, not a single subject in the XTEND-1 trial reported zero bleeds during the on-demand treatment period. Thus, PN = 100%.

The probability of sufficiency (PS) is defined as PS = P (Yx = true | X = false, Y = false). This reflects the probability that administering Altuvoct would result in no bleeding in a situation where the patient did not receive the treatment and did experience bleeding. It quantifies whether the treatment alone is enough to prevent bleeding. Across the studies, 65% of subjects experienced no bleeds while on Altuvoct prophylaxis, indicating PS = 65%.

The probability of necessity and sufficiency (PNS) is defined as: PNS = P (Y = true | do (X = true)) − P (Y = true | do (X = false)). This measures the probability that the outcome occurs if and only if the treatment is administered. It captures the joint effect of necessity and sufficiency.

In the case of severe haemophilia A, if Altuvoct is not administered as prophylaxis, then the probability of no bleeds would be zero, i.e., P (Y = true|do (X = false) equals 0 (as was found during the on-demand phase of the XTEND-1 study); the PNS then becomes equal to P (Y = true|do (X = true)). Based on the data of the submitted studies, subjects with severe haemophilia A no longer experienced any bleeds, with a 65-per cent probability if and only if they were administered Altuvoct (PNS = 65%).

## 4. Discussion

Statistical inference plays a critical role in clinical trials by establishing the efficacy and safety of medicinal products. Among trial designs, randomised, blinded, controlled trials are considered the gold standard for evaluating treatment efficacy. Randomisation helps reduce bias, balances confounding variables, and supports valid significance testing. To ensure statistical robustness, such trials typically involve large participant numbers to generate reliable efficacy data.

However, there are circumstances where randomised controlled trials (RCTs) may not be feasible, particularly due to ethical concerns or practical limitations. In these cases, single-arm trials may be the only available source of evidence [21]. For efanesoctocog alfa, the manufacturer’s key claims were supported by two primary studies, both of which were single-arm, non-randomised, unblinded, and lacking internal control groups [11,13].

While inferential statistics can be used to compare treatment effects between groups, their application in non-randomised, open-label settings does not allow for valid probability statements about causal treatment effects due to an inability to control for bias. Furthermore, single-arm studies cannot support the foundational assumptions of randomisation. Though traditional statistical methods may still be applied in a descriptive capacity to single-arm studies, doing so disconnects the analysis from any causal interpretation of the outcomes [13,15].

To address these limitations, a ‘cause-of-effect’ approach was adopted. This method is based on a structural causal model composed of three key elements: (i) graphical models, representing known relationships in the real world; (ii) structural equations, expressing how variables influence one another; and (iii) counterfactual and interventional logic, capturing what we aim to understand and predict. Together, these components form a framework where graphical models express background knowledge, counterfactual reasoning formulates the causal questions of interest, and structural equations integrate the two into a coherent causal analysis [16,18].

Haemophilia A is caused by a deficiency of functional FVIII in the plasma and leads to frequent episodes of bleeding into joints, soft tissues, and muscles, either spontaneously or following even minor trauma. People with severe haemophilia A require frequent administration of factor VIII supplements to treat episodes of bleeding and to provide prophylaxis. Using data from the two main studies submitted, the cause-of-effect analysis demonstrated that subjects with severe haemophilia A no longer experienced any bleeds, with a 65-per cent probability if and only if they were administered Altuvoct.

In order to confirm that exposure to Altuvoct may be considered as the actual cause of the ideal of zero bleeds in subjects with severe haemophilia A, we employed the three-point actual cause model of Halpern [17,22], where all three points must be fulfilled. The three points are as follows:

Point 1 states that exposure to Altuvoct cannot be considered a cause of zero episodes of bleeding unless both exposure to Altuvoct and zero episodes of bleeding actually happen. In this case, prophylactic administration of Altuvoct and an outcome of zero overall episodes of bleeding both occurred, and so point 1 was fulfilled.

Point 2 requires both necessity and sufficiency and takes the form of a ‘but for’ statement combined with what happens in the actual situation. Thus, given the natural history of severe haemophilia A, all subjects (i.e., 100%) would have bled over the course of the follow-up in the submitted studies, but with the administration of Altuvoct, and, in the course of the studies, 65% of subjects achieved zero overall episodes of bleeding. The necessity and sufficiency conditions occurred, and so point 2 was fulfilled.

Point 3 assumes the minimality condition, wherein the cause does not have any irrelevant or trivial components (any inessential elements are pruned). In this case, we confined our analysis to only the essential elements, as illustrated in the structural causal model in Figure 2, and so point 3 was fulfilled.

Points 1, 2, and 3 of the actual cause model were all found to be fulfilled, and so we concluded that the actual cause of the zero episodes of bleeding in the subjects with severe haemophilia A was the prophylactic administration of Altuvoct.

In this cause-of-effect analysis, we assumed the following: (i) monotonicity, where all individuals were affected in the same direction (a reduction in the episodes of bleeding) or not at all by exposure to Altuvoct, and (ii) that the outcome was generalisable to other settings because severe haemophilia A is caused by the absence of plasma activity of factor VIII, and only compliant exogenous supplementation of the plasma activity of factor VIII will lead to the ability to form blood clots and, thus, prevent episodes of bleeding [23,24].

The limitations of a causal analysis are related to the assumptions [18] of the proposed causal structure and the mechanisms that are displayed in Figure 2. It is acknowledged that these assumptions may not capture the complexity of the real world.

There were no reports of serious allergies or anaphylaxis or thrombo-embolic events in the submitted studies; treatment-emergent anti-drug antibodies were detected in only four subjects and were found to be transient.

Aspects of safety would not be considered to give rise to particular concern at this stage, yet the total number of subjects in the studies was regarded as small, with limited exposure (although the study periods lasted for 52 weeks, subjects in real life may be exposed to this product lifelong). Knowledge of aspects of safety will be investigated via a risk management plan (RMP). The RMP was designed to gather additional data to assess risks which were not fully addressed by the provided data, particularly the long-term safety and efficacy of Altuvoct. The data gathered will also permit additional comparisons with existing standard treatment regimens. According to the Altuvoct RMP, a post-authorisation long-term safety and efficacy study is ongoing, and an observational registry study in previously untreated patients with haemophilia A is planned [25].

Regarding the age of the subjects, the company employed a physiologically based pharmacokinetic (PBPK) analysis to support its claims. The PBPK model incorporated distribution and clearance mechanisms of efanesoctocog alfa and extrapolated from adults to paediatrics, taking ontogeny into account. The modelling exercise indicated that the pharmacokinetic parameters of dosing with 50 IU/kg would be appropriate and acceptable for the paediatric population aged less than 6 years (and the actual pharmacokinetic data for the paediatric participants aged less than 6 years in the XTEND-KIDS study were in line with expectations).

From a regulatory perspective, the PBPK model used in this procedure was considered to have a high regulatory impact as it replaced clinical studies that would normally be conducted to confirm the safety and efficacy of the proposed dose of efanesoctocog alfa in the paediatric population aged less than 2 years. The model served as the primary source of information for the company to propose a paediatric dose for subjects aged less than 2 years, yet this approach was considered to be of high risk for these subjects because of the potential for the models to misinform. As a consequence, the model had to be sufficiently validated with external pharmacokinetic data in order to ensure the accuracy of the model predictions. In this case, however, it was found that there was insufficient validation of the described model with clinical data from paediatric patients aged less than 2 years as well as a lack of sufficient knowledge on ontogeny parameters (such as the expression of the neonatal Fc receptor and its modulation factors) for paediatric patients less than 2 years old, and so the model was not considered sufficient to support the dose selection of Altuvoct in paediatric patients less than 2 years old.

Regarding moderate and mild haemophilia A, the submitted clinical studies were only conducted in patients with severe haemophilia A (FVIII < 1%). Use in subjects with moderate (1–5%) or mild haemophilia A (6–40%) as a prophylactic treatment was questioned because of the extended half-life of 40 h of Altuvoct, which is notably longer than that of currently approved FVIII medicinal products, where the half-life is around 24 h. There was concern that administration to those with moderate or mild haemophilia A would lead to overexposure with subsequent harm.

A simulation exercise provided by the company of steady-state FVIII activity in patients with moderate haemophilia A (5 IU/dL) and severe haemophilia A (0 IU/dL) receiving weekly prophylaxis with efanesoctocog alfa (50 IU/kg) gave assurance that overexposure is unlikely in subjects with moderate haemophilia A (5 IU/dL) and that the percentage fraction above threshold is comparable to that of subjects with severe haemophilia A (0 IU/dL). From this, there were no clinical, pharmacological objections to the use of efanesoctocog alfa (50 IU/kg) in subjects with moderate haemophilia A. A corresponding exercise for subjects with mild haemophilia was unable to assuage concerns, and so the claimed indication was limited to those with severe or moderate haemophilia A only.

In summary, we produced a causal analysis for single-arm studies submitted to make the claim for the prophylaxis of episodes of bleeding in subjects with severe (and moderate) haemophilia A. Such ‘cause-of-effect analysis’ provides an alternative to conventional statistics (frequentist and Bayesian) [14] and, in our opinion, avoids the difficulties of conventional statistics associated with single-arm studies (a low power of study, a lack of randomisation, and a lack of a comparator) that hinder the interpretation of outcomes.

## 5. Conclusions

Overall, the claim that Altuvoct is indicated for the prophylaxis of episodes of bleeding in people with severe or moderate haemophilia A was considered supported by the results of the submitted studies; the benefit–risk evaluation of Altuvoct in the target populations was found to be positive.

## Data Availability

Additional data will be made available upon request to the authors at the following email address: essam.kerwash@mhra.gov.uk.

## Abbreviations

The following abbreviations were used in this manuscript:

PBPK	Physiologically based pharmacokinetics
PNS	The probability of necessity and sufficiency
PN	The probability of necessity
PS	The probability of sufficiency
FVIII	Factor VIII

## References

[1] Bannow B.S., Recht M., Négrier C., Hermans C., Berntorp E., Eichler H., Mancuso M.E., Klamroth R., O’Hara J., Santagostino E. (2019). Factor VIII: Long-established role in haemophilia A and emerging evidence beyond haemostasis. Blood Rev..

[2] Iasmina-Maria I., Emilia S., Alexandra M. (2024). Transforming Hemophilia A Care: Insights into New Therapeutic Options. Life.

[3] Carcao M.D., Chelle P., Clarke E., Kim L., Tiseo L., Morfini M., Hossain T., Rand M.L., Brown C., Edginton A.N. (2019). Comparative pharmacokinetics of two extended half-life FVIII concentrates (Eloctate and Adynovate) in adolescents with hemophilia A: Is there a difference?. J. Thromb. Haemost..

[4] Klamroth R., Kragh N., Arnaud A., Guyot P., Wilson A., Wojciechowski P., Wdowiak M., Margas W., Bystrická L., Tosetto A. (2025). Efanesoctocog Alfa versus Standard and Extended Half-Life Factor VIII Prophylaxis in Adolescent and Adult Patients with Haemophilia A without Inhibitors. Adv. Ther..

[5] Junzheng W., Liu X., Yang H., He Y., Yu D. (2024). Advances in biopharmaceutical products for haemophilia. iScience.

[6] Gualtierotti R., Giachi A., Bitto N., La Mura V., Peyvandi F. (2024). Gene therapy in hemophilia: The dawn of a new era. Res. Pract. Thromb. Haemost..

[7] Malec L., Peyvandi F., Chan A., Koenigs C., Zulfikar B., Yuan H., Simpson M., Alvarez-Román M., Carcao M., Staber J. (2023). (LB 01.1) Efanesoctocog alfa prophylaxis for previously treated patients <12 years of age with severe hemophilia A. Res. Pract. Thromb. Haemost..

[8] von Drygalski A., Chowdary P., Kulkarni R., Susen S., Konkle B.A., Oldenburg J., Matino D., Klamroth R., Weyand A.C., Jimenez-Yuste V. (2023). Efanesoctocog alfa prophylaxis for patients with severe hemophilia A. N. Engl. J. Med..

[9] Altuvoct UK Public Assessment Report. https://mhraproducts4853.blob.core.windows.net/docs/c4e707b0b30603b9c0755e35e0dad26aa9219a10.

[10] Toulmin S., Rieke R., Janik A. (1984). An Introduction to Reasoning.

[11] Toulmin S. (2003). The Uses of Argument, Updated Edition.

[12] Malec L., Peyvandi F., Chan A.K., Königs C., Zulfikar B., Yuan H., Simpson M., Román M.T.Á., Carcao M., Staber J.M. (2024). Efanesoctocog Alfa prophylaxis for children with severe hemophilia A. N. Engl. J. Med..

[13] Statistical Principles for Clinical Trials. ICH E9. https://database.ich.org/sites/default/files/E9_Guideline.pdf.

[14] Pearl J. (2009). Causal inference in statistics: An overview. Stat. Surv..

[15] Greenland S. (1990). Randomization, statistics and causal inference. Epidemiology.

[16] Pearl J. (2015). Causes of effects and effects of causes. Sociol. Methods Res..

[17] Halpern J.Y. (2016). Actual Causality.

[18] Pearl J. (1999). Probabilities of causation: Three counterfactual interpretations and their identification. Synthese.

[19] Pearl J., Glymour M., Jewell N.P. (2016). Causal Inference in Statistics.

[20] Tian J., Pearl J. (2000). Probabilities of causation: Bounds and identification. Ann. Math. Artif. Intell..

[21] Li S., Ling S., Wang D., Wang X., Hao F., Yin L., Yuan Z., Liu L., Zhang L., Li Y. (2024). Modified lentiviral globin gene therapy for pediatric β^0^/β^0^ transfusion-dependent β-thalassemia: A single-center, single-arm pilot trial. Cell Stem Cell.

[22] Halpern J.Y. A modification of the Halpern-Pearl definition of causality. Proceedings of the 24th International Joint Conference on Artificial Intelligence (IJCAI 2015).

[23] Answering Questions Using Observational Data, Published by The Committee for the Prize in Economic Sciences in Memory of Alfred Nobel. 11 October 2021. https://www.nobelprize.org/uploads/2021/10/advanced-economicsciencesprize2021.pdf.

[24] Esterling K.M., Brady D., Schwitzgebel E. (2025). The necessity of construct and external validity for deductive causal inference. J. Causal Inference.

[25] Altuvoct: EPAR—Risk-Management-Plan. https://www.ema.europa.eu/en/documents/rmp-summary/altuvoct-epar-risk-management-plan_en.pdf.

